# Vitamin D: Moving Forward to Address Emerging Science

**DOI:** 10.3390/nu9121308

**Published:** 2017-12-01

**Authors:** Christine L. Taylor, Christopher T. Sempos, Cindy D. Davis, Patsy M. Brannon

**Affiliations:** 1 Office of Dietary Supplements, National Institutes of Health, Room 3B01, 6100 Executive Boulevard, Bethesda, MD 20892, USA; SemposCH@nih.gov (C.T.S.); DavisCI@nih.gov (C.D.D.); 2 Division of Nutritional Sciences, 225 Savage Hall, Cornell University, Ithaca, NY 14853, USA; pmb22@cornell.edu

**Keywords:** vitamin D, data evaluation, cancer, assay standardization, dietary reference values, vitamin D standardization program

## Abstract

The science surrounding vitamin D presents both challenges and opportunities. Although many uncertainties are associated with the understandings concerning vitamin D, including its physiological function, the effects of excessive intake, and its role in health, it is at the same time a major interest in the research and health communities. The approach to evaluating and interpreting the available evidence about vitamin D should be founded on the quality of the data and on the conclusions that take into account the totality of the evidence. In addition, these activities can be used to identify critical data gaps and to help structure future research. The Office of Dietary Supplements (ODS) at the National Institutes of Health has as part of its mission the goal of supporting research and dialogues for topics with uncertain data, including vitamin D. This review considers vitamin D in the context of systematically addressing the uncertainty and in identifying research needs through the filter of the work of ODS. The focus includes the role of systematic reviews, activities that encompass considerations of the totality of the evidence, and collaborative activities to clarify unknowns or to fix methodological problems, as well as a case study using the relationship between cancer and vitamin D.

## 1. Introduction

Vitamin D remains a major research focus. No doubt there is great interest in the question of vitamin D, ranging from the reason for the presence of its receptors in many body cell types, to the relationship between intake and overall health outcomes. Vitamin D is a component of the diet, but it is a unique nutrient in that it functions as a prohormone, which may be endogenously produced under conditions of sun exposure. Regardless if whether obtained from food, supplements, or sun exposure, it functions similarly in the body and must undergo further processing by the body in order to become the physiologically active hormone (1,25-dihyroxyvitamin D). The active hormone has potent cell signaling abilities and is tightly regulated at the tissue level.

Table 1 lists examples of health conditions for which vitamin D involvement has been suggested. The PubMed search engine identifies more than 71,000 publications on vitamin D in general, and at least 14,000 publications that are related to vitamin D and health specifically [1]. Approximately 3000 publicly and privately supported vitamin D clinical studies are listed within the United States (U.S.) government’s Web-based registry of clinical trials [2].

Yet, there remains considerable uncertainty surrounding vitamin D—a nutrient that is characterized by emerging science. Despite much conjecture about its role beyond bone health, the science does not reflect an agreed-upon trajectory for many conclusions about vitamin D. Some have identified vitamin D as a notable nutrient that is responsible for many benefits that are associated with reducing chronic disease risk, and some advocate for greater health through a increased consumption of vitamin D. Yet, others conclude that the interest surrounding vitamin D may be similar to an earlier experience, which is referred to as the “beta carotene phenomenon”, in which supplementation with beta carotene (a precursor of the nutrient vitamin A, and quite distinct from vitamin D) was initially purported to be beneficial and widely recommended. Subsequently—after clinical trials—beta carotene was found to have unanticipated detrimental effects or a spurious benefit. In any case, it is evident that the science surrounding vitamin D is far from settled. At the same time, research continues to accumulate. A key question is how such data are evaluated, enhanced, synthesized, and incorporated into scientific conclusions and related recommendations about vitamin D so that these are reasonably stable over time and ensure public confidence in health guidance. 

The Office of Dietary Supplements (ODS) at the National Institutes of Health (NIH) has a mission to foster dialogue and research related to scientific uncertainties. During recent years it has worked to address vitamin D, both as a supplement and within the context of total exposure. The underlying interest of the office is to encourage the development of systematic reviews along with consideration of the totality of the evidence when reviewing the science surrounding vitamin D. An additional ODS focus has been collaborative activities to support research and foster improved methodologies and scientific understandings. These activities reflect the types of efforts that help to deal with uncertain data, identify knowledge gaps to be addressed, and work to move the science to the next step. 

## 2. Promoting Consideration of the Totality of the Evidence 

### 2.1. Systematic Reviews Underpin the Totality of Evidence

The systematic review process within the field of nutrition has been described by others [4,5]. Briefly, a systematic review is a structured process to comprehensively identify, examine, compare, and synthesize available literature. As such, a systematic review is likely the single best approach to surveying literature in anticipation of drawing conclusions that are based on current evidence. It begins with an analytic framework to guide the formulation and refinement of the questions to be asked of the systematic review. These questions are central to ensuring that the completed systematic review meets the needs of its intended users. An example question might be: “What is the efficacy or association of vitamin D intake levels in preventing incidence CVD (cardiovascular disease) outcomes in people without known CVD (i.e., primary prevention) and with known CVD (i.e., secondary prevention)?” Question formulation is followed by the development of a search strategy, specification of the appropriate inclusion and exclusion criteria for the studies to be examined, and then the determination of a grading system to rate studies based on methodological quality and bias. The systematic review comes to a close at the point when data are extracted and summarized in tables. The final step is the compilation of an evidence report. 

Systematic reviews evolved from the field of clinical medicine, for which questions tend to be more straightforward than issues related to nutritional topics. Nutritional questions are by their nature more complex because there is often a high degree of uncertainty as well as considerable variability among nutrition studies. In short, there may be considerable data on a specific nutrient, but cumulatively the data do not lend themselves to merging for increased statistical power [5]. For these reasons, approaches to developing systematic reviews for nutrition questions, including vitamin D, have required some specification to facilitate a better understanding of the challenges involved and the relevance of the review. Between 2009 and 2013, ODS supported efforts by the Agency for Healthcare Research and Quality (AHRQ) to develop six technical reviews that outlined the application of systematic review methodologies to the field of nutrition [6]. Specifically, for vitamin D, ODS co-funded a 2007 systematic review [7] that focused on bone health that examined specified subpopulations and total exposure levels accounting for vitamin D exposures from diet and synthesis in the skin. This systematic review incorporated intermediate biomarkers and surrogate health outcomes and took into account measures of serum 25(OH)D. A 2009 systematic review on vitamin D that was also co-funded by ODS [8] focused on a range of health outcomes in addition to bone health, again including both RCT and observational studies. It was based on two analytic frameworks—one that addressed deficiency and adequacy relative to the nutrient and the health outcomes, and one that focused on adverse outcomes relative to vitamin D excess or toxicity. In preparation for a 2014 special dialogue about vitamin D [9], ODS funded an update of the 2009 systematic in order to determine whether newer data had changed the conclusions of the 2009 systematic review; the conclusions remained stable when the newer data were taken into account [10]. These systematic reviews have provided a useful platform not only for recommendations about the nature of strength of the relationship between vitamin D and certain health conditions, but also as a way to identify and focus research needs. Table 2 contains examples of the types of data limitations that present challenges in carrying out systematic reviews for vitamin D. 

### 2.2. Incorporating the Totality of the Evidence

The development of nutrient reference values illustrates the nature of the steps that are necessary to ensure that the totality of the evidence is taken into account when making scientific conclusions or underpinning public health recommendations, an approach that is central to the ODS perspective on addressing emerging science. In the United States and Canada, reference values, known as Dietary Reference Intakes (DRIs), are established by expert committees that are convened by the Institute of Medicine (IOM), now the National Academy of Medicine. The approach used by the IOM to establish vitamin D reference values demonstrates a process that takes into account the totality and strength of the evidence [3,11]. The values have key roles in public health policy as well as research activities. 

In 2008, ODS and other federal sponsors co-funded IOM’s efforts to convene an IOM committee to establish DRIs for vitamin D, as well as calcium, with a final report being issued in 2011 [3]. The committee carried out a number of steps to ensure the needed consideration of the totality of the evidence. At the beginning of the IOM committee’s work, it outlined the intended evaluation process, as shown in Table 3. To determine the health outcome that would serve as the basis for the DRI for vitamin D, it next considered the overall literature regardless of the nature of the evidence, including those relationships that are listed in Table 1. The available systematic reviews on vitamin D, as outlined above, were a critical initial aspect of surveying the totality and strength of the available evidence. The committee also recognized that useful studies had been published after the completion of these reviews and that several relevant studies for the committee’s interests did not meet the inclusion criteria for the AHRQ analyses and had not been reviewed. The committee added such data to its deliberations. As a general matter, the committee determined that RCTs provided the greatest level of confidence, but it also considered the outcomes from observational studies. The comprehensive examination of the entire dataset required an evaluation of data quality and strength, identification of consistency of effect, and searches for confounding factors. 

**Figure 1:**
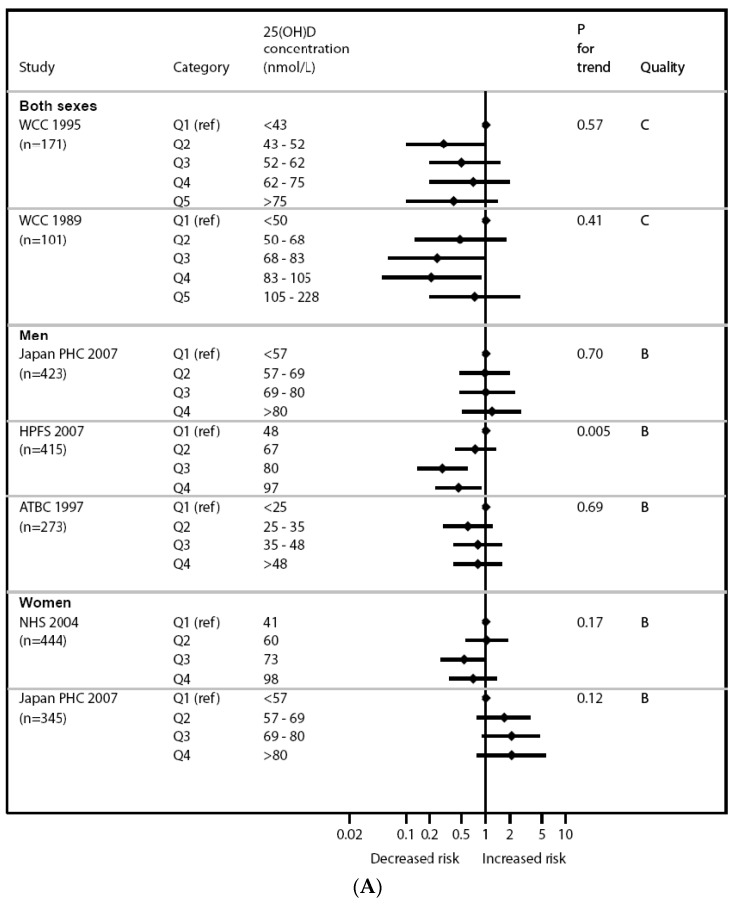

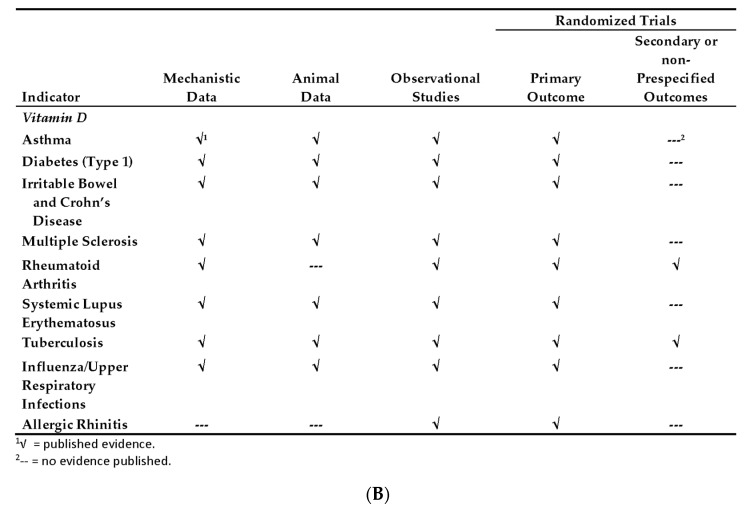
Illustration of data arrays to evaluate totality of the evidence. (**A**) Forest plot for colon cancer risk stratified by vitamin D concentration (from Chung et al., 2009 [8], Figure 9); (**B**) Evidence map for vitamin D and immune outcomes (modified and updated based on IOM, 2011 [3], Table E-5; reproduced with permission). Abbreviations: WCC = Washington County Cohort, Women’s Health Initiative; PHS = Public Health Centers; HFPS = Health Facilities Program Section; ATBC = α-Tocopherol, β-Carotene Cancer Prevention Study; NHS = National Health Survey.

For ease of its consideration of the totality and strength of the evidence, data can be arrayed in a variety of ways. Figure 1A is an example of a forest plot, in this case showing the relationship between vitamin D and colorectal cancer risk, and reflects the nature of the inconsistent results. Figure 1B illustrates an evidence map for vitamin D and immune outcomes, and indicates the limited number of trials. Through this type of detail-oriented approach, the committee determined that the evidence was insufficient to establish a causal link between vitamin D and health outcomes other than bone health. This insufficiency reflected—on balance given the totality of the evidence—three limitations of the evidence: the data failed to demonstrate causality, research outcomes were contradictory, and that the effects were inconsistent across studies [3]. For the identified bone health outcome, the committee integrated the following measures: calcium absorption, bone accretion (including bone mineral content/density and rickets), bone maintenance (including bone mineral density and osteomalacia), and bone loss (including fracture risk).

Using fractures as an example of integration, the types of summaries from systematic evidence-based reviews that were important to the committee’s conclusions are highlighted in Table 4. The evidence for fractures was rated as good, but it could not be characterized as strong. Other evidence that was gleaned by the committee from the AHRQ reviews indicated that calcium absorption was enhanced only when serum 25(OH)D levels were quite low (<12.5 nmol/L); a linear relationship between serum 25(OH)D and calcium absorption could not be demonstrated; relationship between parathryroid hormone and serum 25(OH)D concentrations was too inconsistent to be useful; and, bone mineral content clearly increases with vitamin D exposure in children, but the relationship is not strongly demonstrated in adults.

**Figure 2:**
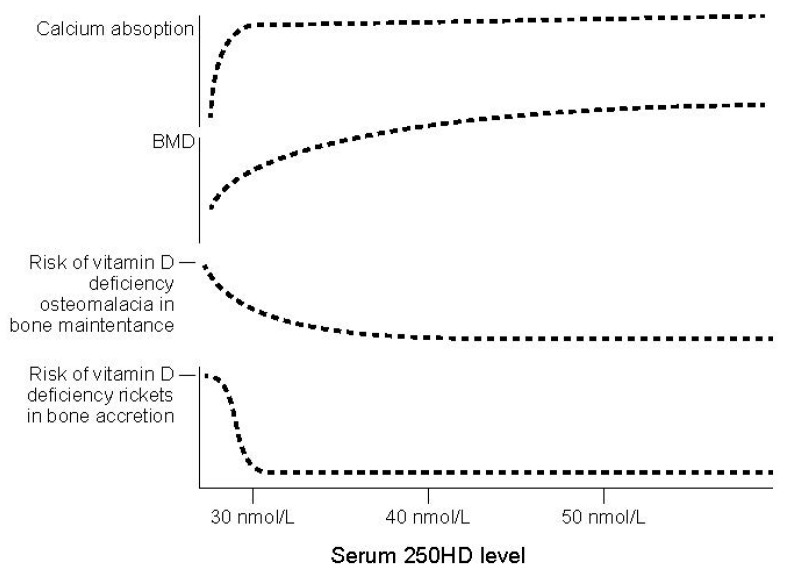
Conceptualization of integrated bone health outcomes and vitamin D exposure (from Institute of Medicine (IOM), 2011 [3], Figure 5-1; reproduced with permission).

The committee next focused on specifying a dose-response relationship between serum 25(OH)D concentrations and bone health based on the identified measures. Compiling and integrating data led the IOM committee to conclude that the strongest evidence linked a concentration of 16 ng/mL (40 nmol/L) serum 25(OH)D to an average requirement for bone health (Figure 2), the value that was established as the Estimated Average Requirement or EAR. The committee had no reason to assume that the requirement for vitamin D was not normally distributed. Therefore, based on a two-standard-deviation calculation, the level that surpassed the need for 97.5% of the population was 20 ng/mL (50 nmol/L), the value established as the Recommended Dietary Allowance or RDA. Although some studies may have suggested a higher average requirement and some studies a lower one, the weight of the totality of the evidence rested on a 16 ng/mL (40 nmol/L) concentration. As an aside, it is worth noting the efforts that are made to make clear that the RDA value of 20 ng/mL (50 nmol/L) does not reflect a clinical cut-point for deficiency, as it is at times misunderstood to be [12].

Finally, the committee specified the total dietary intake that is needed to achieve the EAR- and RDA-linked values for serum 25(OH)D concentrations [3]. Newer studies have enabled linkages to be made between vitamin D intakes and changes in serum 25(OH)D concentrations under conditions of minimal sun exposure, thereby mitigating issues that may be confounded by endogenous production of the substance. These were informative in estimating a dose-response of total vitamin D intake with achieved 25(OH)D concentrations.

A goal of DRI development is to ensure that the conclusions can stand the test of time. That is, reference values should be based on endpoints with data that are not likely to be quickly reversed with a few additional studies. As an example of this concern, at the time of the committee’s work efforts to link the relationship between reduced risk of falls in older individuals and high doses of vitamin D (presumably due to improvements in muscle strength and lower extremity function), was gaining momentum that was based on both observational data and clinical trials. The committee considered that the available evidence was derived from small, underpowered randomized trials, and appeared to be contradictory among the observational data [3,13]. In 2016, well after the committee’s deliberations, a report was published on a trial that was designed to increase concentrations of serum 25(OH)D to 30 ng/mL among home-dwelling seniors (70+ years) to reduce the risk for a repeat fall [14]. It showed the opposite outcome, falls appeared to increase with supplementation. The study concluded that high monthly doses of vitamin D might not be warranted in seniors with a prior fall because of a potentially deleterious effect on falls. 

Reference values are also established by authoritative bodies in other countries, including the Scientific Advisory Committee on Nutrition (SACN) in the United Kingdom and the European Union’s European Food Safety Authority (EFSA). Comparison of the IOM outcomes for vitamin D with those from SACN [15] and EFSA [16] underscores the value of a comprehensive review relative to providing sustainable and consistent conclusions. The three authoritative bodies, using the same available research findings, established reference values that are comparable (or slightly lower) than those that are specified by IOM (Table 5). These expert panels were each charged with considering the totality of the evidence, yet worked independently to use systematic reviews, integrate data, select viable health outcomes related to bone health, conduct special analyses of intake data concomitant with minimal sun exposure, and link a specified dietary requirement to a serum 25(OH)D concentration. The estimates of dose-response and the distribution of serum concentrations of 25(OH)D reflective of bone health were notably consistent and underscored the value of systematic reviews and of incorporating the totality of the evidence. 

Finally, the effort to establish DRIs highlighted data gaps in the field of vitamin D research that are particularly germane to the process of establishing nutrient requirements. Examples of these are shown in Table 6. Notable among them is the need for better dose-response data for vitamin D, an effort to design studies that allow for the effect of vitamin D to be determined separately from the effect of calcium intake, focused research related to the effect of excessive vitamin D and its adverse effects, and further elucidation as to the appropriateness of serum 25(OH)D as a biomarker of effect. Concomitant with these interests is the need to develop methodologies that enhance approaches to conducting systematic reviews that make the best use and appropriate integration of a wide array of data including epidemiological reports, clinical trials and mechanistic studies.

### 2.3. Cancer and Vitamin D: Illustration of Why Further Research Is Worthwhile

Science, of course, continues to advance. An important factor in arguing for further research relative to vitamin D is the notable expansion of interest in the topic given the many receptors in the human body coupled with the mixed nature of available data to date, the number of health benefits that have been hypothesized, the numerous confounding factors that complicate interpretation of the data, and its unique role as a nutrient that is also a hormone. The topic of cancer and vitamin D illustrates the continued progression of understandings that have evolved following the development of DRIs and the need for further research, and, in turn, it serves to reflect similar needs for other vitamin D health relationships. 

The hypothesis that vitamin D might exert cancer protective effects was first suggested 30 years ago by ecologic or geographic correlation studies, which demonstrated lower cancer mortality in regions with greater exposure to solar UV-B radiation [17,18]. Because ultraviolet radiation can result in vitamin D formation in the skin, this led to the hypothesis that vitamin D or one of its metabolites (25(OH)D or 1,25(OH)_2_D) may be protective against cancer. To date, preclinical studies usually have demonstrated the protective effects of vitamin D against cancer through the modulation of many different molecular targets and biological processes that are dysregulated during carcinogenesis, including cell proliferation, apoptosis, inflammation, cell differentiation, angiogenesis, invasion, and metastasis [19]. Similarly, epidemiological studies have generally demonstrated an inverse association of plasma 25(OH)D concentrations with colorectal cancer incidence and mortality [20,21]. In contrast, the association between vitamin D intake and incidence of colorectal cancer is conflicting [21], and the relationship between plasma 25(OH)D concentrations and other cancers is less clear [19]. 

This disparity between the preclinical literature on vitamin D supplementation and cancer and the epidemiologic literature on vitamin D status and cancer can best be resolved by randomized controlled trials investigating the relationship between vitamin D supplementation and cancer. A Cochrane review was conducted to assess the beneficial and harmful effects of vitamin D supplementation for the prevention of cancer in adults [22]. Eighteen randomized trials with 50,623 participants were included in the analyses. Cancer occurrence was observed in 1927/25,275 (7.6%) recipients of vitamin D versus 1943/25,348 (7.7%) recipients of control interventions (RR 1.00 (95% confidence interval (CI) 0.94 to 1.06); *p* = 0.88). The authors of this analysis concluded “There is currently no firm evidence that vitamin D supplementation decreases or increases cancer occurrence in predominantly elderly community-dwelling women [22].” However, the authors also concluded, “We need more trials on vitamin D supplementation, assessing the benefits and harms among younger participants, men, and people with low vitamin D status, and assessing longer duration of treatments as well as higher dosages of vitamin D”.

Internationally, four large (>10,000 participants) randomized controlled trials that are investigating the effect of vitamin D supplementation on the primary prevention of cancer can be found in clinical trial registries (Table 7) [23,24,25,26]. While both *VIT*amin D and Omeg*A*-3 Tria*L* (VITAL) and D-Health have been successful in recruitment [23,24], the Finnish Vitamin D Trial (FIND) was only able to recruit 2500 subjects [25], and it is not clear that the Vitamin D and Longevity (VIDAL) trial is being continued beyond the feasibility study [26].

The VITAL study [23], which was funded by a number of federal agencies, including ODS, highlights how a clinical trial can be used to answer multiple questions and gaps in the literature. VITAL is a randomized double-blind, placebo-controlled 2 × 2 factorial trial of vitamin D and omega-3 fatty acid supplementation for the primary prevention of cancer and cardiovascular disease in a nationwide cohort of 25,874 U.S. adults. This trial includes 5107 African Americans, making it the most racially diverse of the ongoing large randomized controlled trials of vitamin D. 

At baseline, 16,956 participants or 65.5% of the total study population provided blood samples [23]. Blood samples will also be collected at trial years 1–4 from a randomly selected subset of these participants (~6000 subjects). Based on “lessons learned” from the ODS vitamin D standardization program discussed below, the VITAL study investigators are working with two different laboratories that are participating in the CDC vitamin D standardization program to calibrate their assays and analyze the samples for serum 25(OH)D. Moreover, these archived blood samples will serve as a valuable resource to allow for the assessment of effect modification by baseline 25(OH)D concentrations, changes in biomarkers over time, and future genetic analysis. 

In addition to assessing total 25(OH)D concentrations of subjects, the VITAL study is also assessing other potential 25(OH)D-related measures of vitamin D status. The VITAL study investigators plan to use a nested case-control study to measure vitamin D binding protein and free 25(OH)D and to calculate the bioavailable 25(OH)D. It will comprise 2000 incident cases of cancer and cardiovascular disease and 1000 controls, and will assess whether baseline levels of these emerging measures are related to the risk of these outcomes or modify the effect of the vitamin D intervention. Thus, as an important example of efforts to advance understandings about vitamin D, the VITAL study should clarify a number of gaps in the literature, such as whether vitamin D supplementation is protective for the primary prevention of cancer, whether there are ethnic differences in the relationship between vitamin D supplementation and cancer risk, what is the relationship between baseline vitamin D status and the response to vitamin D supplementation and subsequent cancer risk, and what is the utility of evolving biomarkers of vitamin D status. 

## 3. Promoting Research and Collaborative Activities 

### 3.1. ODS Research Portfolio

Efforts both to clarify the basic biological activities of vitamin D and to enhance the applicability of the existing research are often at the forefront of discussions about vitamin D. As funding allows and in collaboration with the institutes and centers at NIH, ODS supports research on topics related to dietary supplements, including vitamin D. Proposals from interested parties are solicited via program announcements, requests for applications, and other mechanisms. A complete listing of ODS co-funded grants can be found on the ODS website [27]. As illustrated by the listing, the topics that are focused on vitamin D are diverse and range from the effect of the nutrient on hyaluronic acid signaling in women with triple negative breast cancer to the effect of vitamin D supplementation on the prevention of falls in the elderly. Grant applications are subjected to a competitive process and are funded on the basis of merit and design quality [28].

Although such grants help to build an important foundation for conclusions about vitamin D, ODS also works to enhance the multiplier effect, which expands and enhances collaborative and follow-on research and activities. This includes dialogues to identify and increase awareness of research needs and knowledge gaps, such as a 2014 conference on evidence-based decision making for vitamin D in primary care [9], as well as long term collaborative projects, such as the program that is focused on standardizing vitamin D measurements as described directly below.

### 3.2. Vitamin D Assay Standardization

Leveraging expertise is an important component of addressing uncertainties, especially given the reality of limited resources. One such ODS-driven collaborative activity is the Vitamin D Standardization Program (VDSP). Currently, there is an international debate regarding the interpretation of serum 25(OH)D concentrations, especially related to questions of dose-response and to determining status [3,15,29]. An important factor contributing to the debate has been assay variability in the measurement of 25(OH)D [30,31]. The lack of standardized assay has made it difficult to pool results from different research studies, which has confounded the development of guidelines for interpreting the concentration of 25(OH)D. These challenges have been a major focus of ODS because serum 25(OH)D is considered to be the best measure of vitamin D exposure and status used to define clinical states, e.g., deficiency, sufficiency, and overload, in nutrition guidelines. 

To address the problem of assay variation, ODS established VDSP in 2010 with the objective to promote the standardized measurement of 25(OH)D worldwide [32]. The VDSP is a public/private partnership that was initiated in collaboration with the Centers for Disease Control and Prevention (CDC), the National Institute for Standards and Technology (NIST), Ghent University, the College of American Pathologists (CAP), the Vitamin D External Quality Assessment Scheme (DEQAS), the ACCC and the International Federation of Clinical Chemistry and Laboratory Medicine (IFCC), national health surveys in Australia, Canada, Germany, Ireland, Mexico, Korea, the United Kingdom, and the US and collaborators from around the world.

A standardized laboratory measurement is one that is accurate and comparable over time, location, and laboratory procedure. In this context, the standardized measurement of 25(OH)D is one in which all of the laboratories using different assays at different times and in different locations obtain the same results—within specified statistical criteria—for the same sample [33]. Moreover, it would be the true concentration as measured by the NIST, Ghent University, and CDC reference measurement procedures (RMP) [34,35,36]. The effect of standardization is that research results from different studies can be pooled—all based on the true concentration of 25(OH)D. This helps to promote the development of evidenced-based guidelines and informed decision making by physicians, policy makers, and patients.

**Figure 3:**
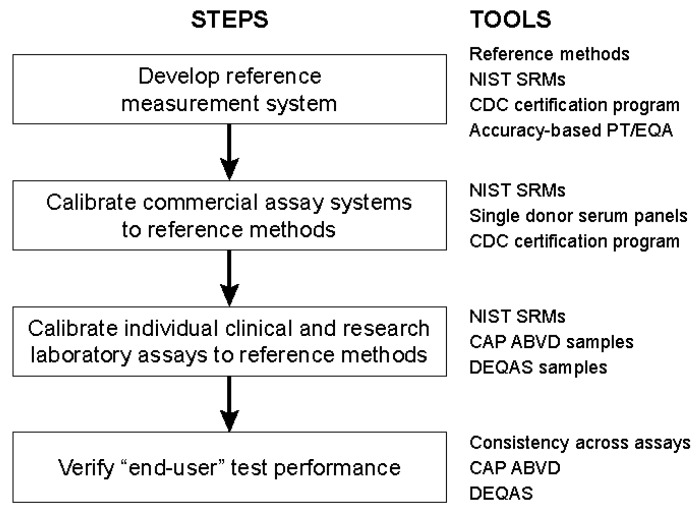
Standardization process for assays of serum 25-hydroxyvitamin D.

To accomplish the goals of standardization, the VDSP, sponsored by NIH/ODS, has worked with its collaborators to assemble a set of tools that can be used to standardize 25(OH)D measurements now and in the future [33]. Those tools include the NIST, Ghent University and CDC RMPs, NIST Standard Reference Materials (SRMs), the CDC Vitamin D Standardization Certification Program (VDSCP), the CAP Accuracy-Based Vitamin D (ABVD) Survey, DEQAS, and statistical criteria to define where standardization exists. SRMs include serum-based materials with target values that are assigned using the NIST RMP and calibration solutions in ethanol (Table 8) [37]. Sets of single donor serum samples with RMP target values can be used in a CDC VDSCP program to standardize commercial assays and large commercial laboratories. In addition, accuracy-based performance testing schemes of the CAP ABVD and DEQAS use materials with RMP target values assigned. The basic steps to the standardization are then to use the tools that are listed to establish a chain of calibration from the RMPs/SRMs to commercial assay manufacturers and then to individual laboratories (Figure 3). Finally, laboratories participating in the CAP ABVD and DEQAS individual laboratories can test if an assay in their use is properly calibrated to the RMPs or traceable. With this VDSP system, individual laboratories can be standardized, again, through a series of calibration steps so that the assay measures the true concentration of 25(OH)D.

At these point standardization programs nearly always stop. However, given the wealth of published data, ODS and its collaborators considered it important to standardize 25(OH)D data from national health surveys, clinical trials, and other key completed studies where properly banked serum samples exist [38,39]. Thus, the VDSP has developed methods that can be used to standardize 25(OH)D measurements from completed studies. Those methods have been successfully used to standardize national nutrition survey data from Ireland [40], Canada [41], and Nordic countries [42], as well as the US NHANES [43]. Newer more cost-effective methods for standardizing completed studies were recently suggested by Jakab et al. [44]. Those new methods are based using small subsets of DEQAS and CAP ABVD materials to determine an equation to calibrate the original 25(OH)D values to NIST-Ghent-CDC RMP standardized or true values [45]. Over time, it is hoped that data from key clinical trials and other research studies will be standardized and added to the pool of true results.

## 4. Implications 

In any field of study, emerging science with its surrounding uncertainties will offer an array of challenges relative to interpretation and application in the public health arena. Perhaps one of the most important lessons to be learned in addressing emerging science is the need to be clear about its nature—its strengths and weaknesses—and to be clear about what is needed to clarify the questions that it raises. The natural tendency to rush to strong conclusions should be avoided when based on limited or contradictory data that are available or on data that are appealing. Rather, the goal should be to make the best conclusions given the totality of the evidence that is appropriately caveated and presented in a transparent manner. Likewise, the desirable research strategy is to actively identify the specific data gaps and systematically work to close them. Vitamin D—and the work of the ODS—offers examples of how such strategies can be employed.

In addition to those data gaps identified in Table 6, there are larger questions, such as how multiple environmental and/or genetic risk factors may interact to produce specific diseases, and, in turn, the relevance to questions of vitamin D in such a scenario. As another example, given the complexity of chronic disease considerations, there is interest in a focus on the cumulative effects of poor health or other disease conditions. Others have noted that the relationship between serum 25(OH)D and rickets due to vitamin D deficiency is used to define cut-points for vitamin D deficiency, or more generally, hypovitaminosis D. This approach is limited by the fact that there are currently inconsistent case definitions of vitamin D in the context of nutritional rickets, measurement is not standardized, and there is inconsistent evaluation of other possible risk factors for nutritional rickets. It may be worth pursuing the establishment of a rickets registry to correct these problems and model the risk of nutritional rickets as a multifactorial disease that is similar in concept to coronary heart disease. Further, research is needed that is related to life-long exposure to vitamin D versus considerations of so-called “snap shot” status measures. Of special interest in order to ensure accurate and relevant measurement of vitamin D—which in turn assists with the ability to combine data from a variety of research sources and to determine status—is the continued focus of the VDSP. For instance, the metabolized and free forms of vitamin D are currently the subject of intense research to determine their role in assessing vitamin D status. Their measurement in vitamin D research should be standardized and harmonized to prevent a recurrence of the problems due to assay variation that has been historically experienced with serum 25(OH)D. 

## Figures and Tables

**Table 1:** Health/disease conditions suggested as linked to vitamin D ^1^.

Cancer/neoplasms including breast, colorectal, prostate Cardiovascular diseases and hypertension Type 2 diabetes Metabolic syndrome (obesity) Falls and physical performance Immune responses including asthma, autoimmune (eczema, type 1 diabetes, inflammatory bowel and Crohn’s disease, multiple sclerosis, rheumatoid arthritis, systemic lupus erythematosus), and mortality due to infectious diseases including tuberculosis and influenza/upper respiratory infections Neuropsychological functioning including autism, cognitive function, and depression Preeclampsia of pregnancy, preterm birth, low birth weight, and infant mortality Skeletal health

^1^ Modified from IOM, 2011 [3], Table 4-1; reproduced with permission.

**Table 2:** Notable evidence gaps for vitamin D.

Many study protocols administer combination of vitamin D and calcium, reducing ability to determine effects of vitamin D independently Data are lacking to examine effects of graded doses to elucidate dose-response relationships Elucidation of mechanisms related to adequate calcium intake diminishing need for vitamin D for bone health Study protocols to address vitamin D as a prohormone with feedback loops related to health effects Additional studies to address effects and nature of sun exposure and ability to integrate sun exposure with intake Determination of the validity of serum 25(OH)D measures as biomarkers of effect Characterization of the variability surrounding measures of serum 25(OH)D concentrations due to different analytical methodologies used Clarification of non-linear relationship between serum 25(OH)D concentrations and increasing vitamin D exposure

Abbreviations: 25(OH)D, 25-hydroxyvitamin D.

**Table 3:** Evidence evaluation components ^1^: Dietary Reference Intake review for calcium and vitamin.

Full review of all purported health outcomes Focus on risk reduction in generally healthy populations Consideration of totality of the evidence ○ Evidence maps as qualitative consideration ○ Summary tables of data arrays ○ Forest plots as appropriate
Strength of evidence to be based on analytic approach, target population, study design and overall quality ○ Consistency of effect ○ Confounding factors ○ Randomized controlled trials reflective of primary outcomes reflect strongest evidence and establish causality and therefore offer higher confidence ○ Lower confidence in observational studies but these are taken into account as confirmatory and to ensure consistency of data.

^1^ Based on discussions in IOM, 2011 [3].

**Table 4:** Evidence for relationship between vitamin D exposure and fractures: Example of good-but-not-strong evidence ^1^.

Outcome	Evidence
Dose-response for fractures	No data
Incidence total fractures: Vitamin D ± calcium vs. placebo	14 RCTs: OR = 0.90 (0.80–1.20)
Incidence total fractures: Vitamin D + calcium vs. placebo	8 non-RCTs: OR = 0.87 (0.76–1.00)
Incidence hip fractures: Vitamin D + calcium vs. placebo	8 non-RCTs: OR = 0.87 (0.76–1.00)

^1^ Compiled from data presented in Chung et al., 2009 [8]. Abbreviations: RCT, randomized controlled trial; OR, odds ratio.

**Table 5:** Comparison of vitamin D reference values and reported approach: Institute of Medicine ^1^, Scientific Advisory Committee on Nutrition (United Kingdom) ^2^ and European Food Safety Authority ^3^.

	IOM	SACN	EFSA
Serum-linked reference value ^4^	EAR: 16 ng/mL RDA: 20 ng/mL	EAR (cannot establish) RNI ≥10 ng/mL	AR (cannot establish) PRI (cannot establish) AI: 20 ng/mL
Intake reference value ^4^	EAR: 400 IU (10 µg) RDA: 600 IU (15 µg)	RNI: 400 IU (10 µg)	AI: 600 IU (15 µg)
Selected Outcome	Skeletal health	Musculoskeletal health	Musculoskeletal health
Components of Selected Outcome	Integrated BMC/BMD, rickets, osteomalacia, calcium absorption, fractures	Rickets, osteomalacia, bone health indicators, fractures, falls, muscle health	Consideration of increased risk of adverse musculoskeletal outcomes
Other Health Outcomes Reviewed But Not Selected	Cancer, diabetes, CVD, falls, immune function, infectious disease, neuropsychological outcomes, pregnancy outcomes	Pregnancy/lactation outcomes, cancer, CVD, hypertension, all-cause mortality, immune modulation, neuropsychological outcomes, oral health, macular degeneration	Pregnancy outcomes, cancer, CVD, immune function, neuropsychological function
Rationale for Non-Selection	Contradictory, inconclusive, lack of causality	Weak, inconclusive	Inconclusive, weak or lacking causality

^1^ IOM, 2011 [3]; ^2^ SACN, 2016 [15]; ^3^ EFSA, 2016 [16]; ^4^ Persons 1–70 years. Abbreviations: IOM, Institute of Medicine; SACN, Scientific Advisory Committee on Nutrition; EFSA, European Food Safety; EAR, Estimated Average Requirement; AR, Average Requirement; RDA, Recommended Dietary Allowance; RNI, Reference Nutrient Intake; PRI, Population Reference Intake; AI, Adequate Intake.

**Table 6:** Examples of research gaps identified during development of Dietary Reference Intakes for vitamin D ^1^.

Research Topic Area	Research Needs
Health outcomes and related conditions	Clarify threshold effects of vitamin D on skeletal health outcomes by life stage and for different racial/ethnic groups. Elucidate inter-relationship between calcium and vitamin D, and specify independent effect(s) of each. Elucidate effect of genetic variation, including that among racial/ethnic groups, and epigenetic regulation of vitamin D on development outcomes.
Adverse effects, toxicity, and safety	Develop innovative methodologies to identify and assess adverse effects of excess vitamin D. Elucidate adverse effects of long-term, high-dose vitamin D. Further explore nature of vitamin D toxicity.
Basic physiology and molecular pathways	Examine the influence of calcium and phosphate on the regulation of vitamin D activation and catabolism through parathyroid hormone and fibroblast-like growth factor 23. Clarify 25(OH)D distribution in body pools including storage and mobilization from adipose tissue. Clarify extent to which differences exist between vitamin D_2_ and D_3_.
Synthesizing evidence and research methodology	Explore enhanced methodologies for data synthesis. Identify approaches to better weight potential health outcomes.
Dose-response relationship	Conduct studies to identify specific health outcomes in relation to graded and fully measured intakes of vitamin D and calcium. Clarify influence of age, body weight, and body composition on 25(OH)D levels in response to intake/exposure.
Sun exposure	Investigate whether a minimal-risk ultraviolet B radiation exposure relative to skin cancer exists that also enables vitamin D production. Clarify how physiological factors such as skin pigmentation, genetics, age, body weight, and body composition influence vitamin D synthesis. Clarify how environmental factors such as sunscreen use affect vitamin D synthesis.
Intake assessment	Enhance dietary assessment methods for vitamin D and calcium intake, and methods for measurement of in foods and supplements. Investigate food and supplement sources for bioequivalence, bioavailability, and safety. Improve standardization of assay for serum 25(OH)D.

^1^ Based on discussions in IOM, 2011 [3].

**Table 7:** Current Randomized-Controlled Trials with >10,000 Participants Investigating Vitamin D Supplementation and Cancer Listed in Clinical Trial Registries.

Trial	Location	Sample Size	Treatment Duration (Year)	Vitamin D Intervention	Primary Endpoints	Trial Registry No.
*VIT*amin D and Omeg*A*-3 Tria*L* (VITAL) [23]	The United States	25,874	5	2000 IU/day	Cancer, Cardiovascular	NCT 01169259
D-Health [24]	Australia	21,315	5	60,000 IU/month	Total mortality, Cancer	ACTRN 1263000743763
Finnish Vitamin D Trial (FIND) [25]	Finland	18,000 ^1^	5	1600 or 3200 IU/day	Cancer, Cardiovascular	NCT 01463813
Vitamin D and Longevity [26] (VIDAL)	United Kingdom	20,000 ^2^	5	100,000 IU/month	Total mortality, Cancer	ISRCTN 46328341

^1^ Projected sample; final randomized sample = 2495; ^2^ Projected sample; status of trial is pending.

**Table 8:** Standard Reference Materials from the National Institute for Standards and Technology.

● Vitamin D metabolites in human serum/plasma
○ SRM 972a Vitamin D Metabolites in Frozen Human Serum
○ SRM 1950 Metabolites in Human Plasma
○ SRM 968e Fat Soluble Vitamins, Carotenoids, and Cholesterol in Human Serum
○ SRM 2973 Vitamin D Metabolites in Frozen Human Serum
● 25-hydroxyvitamin D calibrating solutions in ethanol
○ SRM 2972a
